# High-Quality Draft Genome Sequence of “*Candidatus* Methanoperedens sp.” Strain BLZ2, a Nitrate-Reducing Anaerobic Methane-Oxidizing Archaeon Enriched in an Anoxic Bioreactor

**DOI:** 10.1128/genomeA.01159-17

**Published:** 2017-11-16

**Authors:** Stefanie Berger, Jeroen Frank, Paula Dalcin Martins, Mike S. M. Jetten, Cornelia U. Welte

**Affiliations:** a Department of Microbiology, Institute for Water and Wetland Research, Radboud University, Nijmegen, The Netherlands; b Department of Microbiology, The Ohio State University, Columbus, Ohio, USA

## Abstract

The high-quality draft genome of “*Candidatus* Methanoperedens sp.” strain BLZ2, a nitrate-reducing archaeon anaerobically oxidizing methane, is presented. The genome was obtained from an enrichment culture and measures 3.74 Mb. It harbors two nitrate reductase gene clusters, an ammonium-forming nitrite reductase, and the complete reverse methanogenesis pathway. Methane that escapes to the atmosphere acts as a potent greenhouse gas. Global methane emissions are mitigated by methanotrophs, which oxidize methane to CO_2_. “*Candidatus* Methanoperedens spp.” are unique methanotrophic archaea that can perform nitrate-dependent anaerobic oxidation of methane. A high-quality draft genome sequence of only 85 contigs from this archaeon is presented here.

## GENOME ANNOUNCEMENT

We extracted DNA from a bioreactor highly enriched in “*Ca*. Methanoperedens sp.” strain BLZ2 using the cetyltrimethylammonium bromide (CTAB) method as described previously (1) and the Power soil DNA extraction kit (Mo Bio Laboratories, Uden, The Netherlands). To shear genomic DNA and add adapters in the same step (“tagmentation”), the Illumina Nextera XT library prep kit (Illumina, San Diego, CA, USA) was used. The library was normalized to 4 nM and sequenced with a MiSeq instrument (Illumina) using the 300 paired-end sequencing protocol. Sequencing data were quality trimmed, yielding 11.8 million paired-end reads with an average length of 268 bp. Reads were assembled *de novo* (CLC Genomics Workbench v9.5.2 [CLC bio, Aarhus, Denmark]; word size, 55; maximum bubble size, 5,000). Contigs were binned using MetaBAT (2), yielding a draft genome composed of 85 contigs (*N*_50_ = 74.304 bp). Afterward, quality was assessed using CheckM (3). Annotation was done using Prokka (4) and also as previously described (5). Based on the presence of 228 specific marker genes, the draft genome was 99.35% complete and only 4.58% contaminated. The final sequence had a GC content of 40.3% and was composed of 3.74 Mb with 4,041 putative open reading frames.

The results of this study were largely in accordance with previous data (6, 7). The 16S rRNA gene in this genome was 100% identical to that found in “*Ca*. Methanoperedens” BLZ1 and 95% identical to “*Ca*. Methanoperedens nitroreducens” ANME2d. All genes necessary for reverse methanogenesis were present in the genome, including *mer* (encoding F_420_-dependent 5,10-methenyl-H_4_MPT reductase).

Electron carriers reduced during reverse methanogenesis include coenzyme B (CoB), coenzyme M (CoM), F_420_, and ferredoxin (Fd). It has been suggested that HdrABC/FrhB catalyzes the confurcation of electrons from CoM-SH, CoB-SH, and Fd_red_ to F_420_ (7). Interestingly, in the present data set, four copies of *hdrA* were identified, two of which were fused with *mvhD* (see also reference 8). Furthermore, a gene cluster was identified that contains *hdrABC* as well as *frhB* and two copies of *mvhD*, with *mvhD* possibly being involved in electron transfer.

Membrane-bound enzymes included Ech hydrogenase (*echABCEF* missing *echD*) and the membrane-bound heterodisulfide reductase (*hdrDE*). Furthermore, the complete F_420_:quinone oxidoreductase (Fqo), a Rieske/cyt_b_ complex, and an ammonium-forming cytochrome *c* nitrite reductase (*nrfAH*) were present. Nitrate reductase was present as canonical (NarGHI) and noncanonical (NarGH, potential membrane anchors HCOIIa, HCOIIb, NapH, cytochrome subunit Orf7 and chaperone NarJ, being the similarity to “*Ca*. Methanoperedens” BLZ1 between 92 and 100%) versions, both encoded in two clusters present in close proximity to each other on the same 129 kb-long contig.

### Accession number(s).

This whole-genome shotgun project has been deposited at DDBJ/ENA/GenBank under the accession number NTMG00000000. The version described in this paper is version NTMG01000000.
